# Sustainability of Tailored Goal Oriented Community Brief Intervention Model among Risky Drinkers in Community in Thailand

**DOI:** 10.1155/2013/459402

**Published:** 2013-07-17

**Authors:** Chitlada Areesantichai, Usaneya Perngparn, Catherine Pilley

**Affiliations:** WHO Collaborating Centre for Research and Training in Drug Dependence (WHOCC), College of Public Health Sciences, Chulalongkorn University, Bangkok 10330, Thailand

## Abstract

Tailored Goal Oriented Community Brief Intervention Model (TGCBI), first implemented as culturally secure and acceptable to communities in Thailand, is designed in 2 stages or levels: community level, a culturally secure approach to motivate participants to reconsider their drinking behavior; individual level, involved in the key messages received from the community level together with additional input focused towards individuals. TGCBI's effectiveness was measured by the number of abstinent drinkers and number of alcohol-free months among those who continued to drink at followup in two communities that originally had high prevalence of risky drinking. Multivariate Poisson regression was used to investigate the intervention effect. Results indicated that the number of participants who stopped drinking 6 months later and the number of alcohol-free months during followup were significantly greater (*P* < 0.05) for 47 participants in the intervention group compared to the control group (*n* = 50). TGCBI results in sustainable drinking cessation.

## 1. Introduction

 According to the World Health Organization (WHO), Thailand was ranked 40th in the world statistics on per capita alcohol consumption in 2001 [1]. 

 The 2007 Thai National Household Survey on Substance and Alcohol Use was based on a representative sample as described elsewhere [2]. The principal objective of the survey was to estimate the number of people in Thailand who ever used drugs, including alcohol, tobacco, illicit drugs, and some prescription drugs. This survey used a multistage sampling scheme. The final sampling unit was the household. All residents aged 12–65 years who had lived in that household for more than three months were selected. A face-to-face structured interview was administered by a trained interviewer to elicit data on experiences of substance use in lifetime, past year, and past 30 days. The survey response rate was 84.6%. The results revealed that the past year alcohol consumption was 13.23 million people—28.4% of the population aged 12–65 years, and the past 30 days was 10.54 million—22.7% of the population aged 12–65 years. In addition, more than half of the latter were regarded as current drinkers continuing to drink in the “last week.” Among the 13.23 million who drank during the past year, Alcohol Use Disorders Identification Test (AUDIT) scores indicated that almost 23% (approximately 2.79 million) were “hazardous drinkers,” while 3% (0.39 million) were categorized as “harmful drinkers” and a further 2% (0.23 million) were “alcohol dependent” [2].

During 1992–1996, we studied the alcohol consumption behavior in Lopburi Province and found that the popular alcohol beverages were white spirit, Thai liquor, beer, local liquor, and Chinese liquor, respectively [3]. This study based on Thai National Household Survey in 2007 which indicated Lopburi had the highest alcohol consumption. From 11 districts in Lopburi province, we selected two districts with high risk prevalence of alcohol consumption, that is, Chai Badan district (15.60%) and Phatthana Nikhom district (12.52%) as the control and intervention communities, respectively [2]. This suggests that there is a need to develop and evaluate the impact of strategies to reduce alcohol related harm in such communities.

Brief Interventions (BI) have been identified by WHO as short, simple, and cost effective [4, 5] reducing alcohol consumption among risky drinkers, with direct relevance for a middle income country such as Thailand [6–9]. They can potentially be adopted for implementation within communities.

In Thailand, there are no reports describing community based alcohol interventions. Hence, the Tailored Goal Oriented Community Brief Intervention Model (TGCBI) is the first reported approach to be implemented for risky drinkers in the community to prevent and reduce alcohol dependence. In prior reports of this study, we have described the TGCBI, analyses of AUDIT scores [10], and analyses of reducing alcohol consumption [11]. This paper aims to describe an analysis of the impact of the intervention on drinking over a 6-month followup among risky drinkers in two high drinking prevalence communities. 

## 2. Materials and Methods

The materials and methods detailed of the TGCBI are presented in the previous report [10, 11]. Our study is based on the 2007 Thai National Household Survey. We focused on Lopburi Province which has 11 districts and classified the districts by using the percentage prevalence of alcohol consumption in each district and then categorized as high (more than 10%), medium (6%–10%), and low risk (less than 6%). We selected two high prevalence districts, that is, Phatthana Nikhom (12.52%) and Chai Badan (15.60%) as intervention and control, respectively. We selected two communities that had similar demographic characteristics but are sufficiently distant apart (approximately 100 kms) to reduce contamination across the two sites. We interviewed all participants who met inclusion criteria from every household in the intervention and control groups. We selected only “risky drinkers” (i.e., those identified as hazardous and harmful drinkers) who reported AUDIT scores of 8–19 [12]. 

In our research, we used a quasi-experimental design for two reasons. First, this study selected only risky drinkers who reported AUDIT 8–19 scores [12]. Second, this intervention was implemented in a community. The people who lived in the community know each other. It is very hard to randomize samples without concern of cross-group contamination. 

### 2.1. Study Participants

#### 2.1.1. Number of Participants

 We used a two-tailed test, at 5% significance level, with 80% power, the H_0_ is 5% or less; the target of the intervention was 30%. After calculation, this study required a sample size [13] of 42 for each group. In anticipation of 10% loss for the followup, the sample size was increased to 46.2. All data were analyzed at a single point at the end of the trial. The final study samples were at least 46 participants per group. Two teams of staff interviewed all eligible participants in both intervention and control groups at the same time period. The intervention and control communities are alike in type of alcohol consumption, the number of alcohol shops, and community resources (such as each having a provincial hospital, sanitarium, and temple). Located at a distance of 100 kms from each other (to reduce contamination across the two sites), their alcohol consumption rates were relatively similar.

#### 2.1.2. Inclusion/Exclusion Criteria

 Briefly, the participants in our study were eligible for face-to-face interviews if they were 19 to 65 years old, had lived in the household for more than 6 months, had no recorded history of alcohol dependence, psychiatric disorder or psychosomatic symptoms, and alcohol-related diseases from hospital, and no current pregnancy. The samples were collected from all members of two high-risk drinking communities. In short, they were stable residents, who had symptoms of risky drinking but not evidence of severe dependence or severe related problems. 

#### 2.1.3. Recruitment Strategy

**Figure 1:**
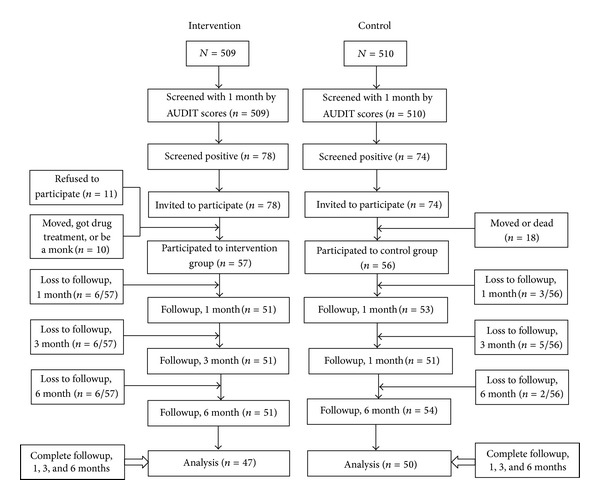
Flow chart of recruitments in each study condition.

 We interviewed all participants who met inclusion criteria from every household in both communities. 509 potential participants were identified for the intervention group and 510 for the control group. Participants studied were selected on the basis of positive screening scores (hazardous drinking scores = 8–15 and harmful level = 16–19) as risky drinkers on the AUDIT [12]. Among 78 risky drinkers from Phatthana Nikhom, 8 participants could not be included: 2 entered monkhood, 1 was undergoing methamphetamine treatment, and 5 were transferred to work in other provinces. Among 70 remaining risky drinkers, 57 participants voluntarily participated but only 47 participants completed the follow-up process and are included in these analyses. Regarding the 74 risky drinkers in Chai Badan, 1 died, 1 entered monkhood, and 16 were transferred to work in other provinces. Among 56 remaining risky drinkers, 50 participants completed the follow-up process and were included in these analyses (Figure 1). The completed follow-up rates were 60.3% and 67.6% in the intervention and control groups, respectively.

### 2.2. Tailored Goal Oriented Community Brief Intervention Model (TGCBI)

 The TGCBI has been described previously [10, 11]. Briefly, we designed TGCBI for our intervention in 2 levels: community/public level and individual level. 

 For the community/public level, we arranged a meeting with key informants (e.g., the abbot, community doctor, and village leader) before the individual level was implemented. Thai communities are strongly influenced by elders, doctors, and monks. It was considered critical that, for the intervention to receive community support, these key individuals were actively engaged in the study. The use of these “key informants” added a unique aspect to the brief intervention which is usually individual focused. Culturally, strong community support for behavior change is an important element of commitment, especially in small Thai communities where people tend to have strong relationships and connectedness across the whole village. A cohesive community with belief in religion and trust in monks, doctors, and village leader and respect for this leadership in the community demanded that the intervention link individually focused brief interventions to a community engagement. TGCBI is an intervention focused not only at private or individual level but also at a community/public level. 

 At the individual level, elements of the brief interventions (BI) as FRAMES were used [4, 5] emphasizing additional input suitable for the community and its cultural context, which key messages received from the community/public level. Below is the additional input.

*Feedback.* We request the participants to assess the level of drinking and related outcomes. Subsequently we provided feedback of the AUDIT scores.

*Responsibility.* We encouraged participants to focus on factors that influenced their alcohol consumption and related harms. They will be inspired to voluntarily commit to change drinking behavior and to participate in our TGCBI.

*Advice.* The participants were provided with advice about how drinking can contribute to problems they experienced and how changing drinking behavior can result in a reduction in problems.

*Menu of Options.* We encouraged the participants to set their own goal(s) but with the participation of family members and/or peers and/or the village leader and/or a monk to help them to achieve their goal(s). This is consistent with Thai culture and community life.

*Empathy.* At the beginning of the TGCBI introduction, the researchers emphasized their understanding of the individual's personal circumstances, the challenges of change, the importance of family and community while building commitment to the intervention, and involvement in the research program.

*Self-Efficacy.* The interval sessions at one, two weeks, and one month of the TGCBI were planned to support the participants' confidence in reducing or abstaining from drinking. The researchers took a role in observing the participants, and progress was monitored, including giving supportive feedback to achieve their goals.

Detailed procedures are provided in previous reports [10, 11]. Operationally, the TGCBI was administered in 4 sessions over 2 months for each participant. Each session took 15 to 60 minutes. All sessions were conducted by the PI (the interventionist) and a counselor. Sometimes a key informant joined one or more sessions. Session 1 was devoted to encouraging the participant by boosting his/her confidence, setting drinking reduction goals, instilling responsibility, and obtaining his/her commitment to reduce drinking. Session 2, about 1 week after session 1, was conducted by monitoring the participant's drinking and ascertaining his/her adherence to drinking reduction goal(s). Session 3 was about 2 weeks after session 2, and the participant had the same goal(s), in addition to providing encouragement, confidence building, and enhancement of self-efficacy. Session 4 was about 1 month after, and the participant had the same goal(s) as session 3. 

Fifty-seven participants (73.1%) in the intervention group volunteered to attend the TGCBI intervention. The proportions of participants attending 1 session, 2 sessions, 3 sessions, and all 4 sessions were 8.8%, 8.8%, 14%, and 68.4%, respectively; that is, more than 82% of participants attended three or four sessions, indicating good adherence to the intervention.

### 2.3. Measurement and Instruments

#### 2.3.1. Measurement

Two separate groups of 5 interviewers collected data in each community at the same time period. They were trained by the PI and not allowed to start interviewing until a level of proficiency was reached. Fidelity to the protocol was monitored by the interventionist. Interviewers were blinded to the design. Consent was obtained at face-to-face interviews using a protocol approved by the Health Sciences Review Board Committee, Chulalongkorn University, Bangkok. 

#### 2.3.2. Instruments

The content of the questionnaire includes demographic characteristics, alcohol consumption, AUDIT and the timeline follow-back instrument (TLFB). The AUDIT's sensitivity and specificity is 0.92 and 0.94, respectively [14]. Additionally, we established validity and test-retest reliability in our community context (Kappa .852). The TLFB [15] is a valid and reliable method of quantifying patterns of alcohol use (30 days) and has been used in a number of investigations to identify intervention outcome in terms of daily alcohol use over extended time periods [16]. The questionnaire was translated into Thai and back-translated into English to ensure its accuracy in translation. 

### 2.4. Measurement of Number of Abstinent Participants and Number of Alcohol Free Months at the Whole 6-Month FollowUp

 The measurement to check the effectiveness of TGCBI outcomes are abstinence for the whole 6-month followup and, for participants who did not achieve full 6-month abstinence, number of alcohol free months at the whole follow-up period. Outcome was analyzed using multivariate Poisson regression rate ratios estimation. Number of alcohol free months at the whole follow-up period outcome is defined as the participants in the intervention and control groups who stopped drinking for a month or more during the whole follow-up period. This was analyzed by Poisson regression. Thus, two drinking outcome measures were used: stopped drinking for the whole 6-month followup and total number of alcohol free months over the whole follow-up period. 

### 2.5. Analysis

 Our study used multivariate analysis: Poisson regression to analyze abstinence for the whole 6-month followup and number of alcohol free months for participants who completed followups at 1, 3, and 6 months in the intervention and control groups. The control variables were current age and gender. The multivariate analysis focused on the effect of the intervention group and control group and aimed to reduce some confounding factors which may affect the outcome variables. Poisson model has a strong theoretical assumption that the conditional variance of the outcome variable equals the conditional mean. Poisson regression gives maximum rate ratios estimation. We obtained robust standard errors for the parameter estimation. We present the regression results as rate ratios. These rate ratios values are equal to coefficients values. All statistical analyses were undertaken using the Stata Program version 11. 

## 3. Results

 When comparing the community demographic characteristics of those who completed all followups in the intervention and control groups (intervention group = 47 participants and control = 50 participants), it was found that they had similar demographic and drinking characteristics, that is, 85.1% and 88.0% were males, and mean ages were 43.3 and 40.7 years among intervention and control participants; and mean of baseline AUDIT scores were 11.6 and 10.8, and percentage of heavy drinking (5 + standards drink) monthly was 42.6% and 42.0% in the intervention and control groups, respectively. Moreover, the participants who completed intervention and control groups were not different from the participants lost to followup. 

 The analysis of intervention on the rate ratios of being abstinent drinking for the whole 6-month followup shows that males were more likely to be abstinent compared to females. Older participants were more likely to be abstinent compared to the younger. Participants in the intervention group were more likely to be abstinent over the whole 6-month followup than the control group. Approximately, 23.4% (11 participants) in the intervention group were abstinent compared to 6% (3 participants) in the control group (Table 1).

Table 2 shows the effect of intervention on total alcohol free months. The results show that males had more alcohol-free months than females, and older participants had more alcohol-free months than the younger participants. In the intervention group, the average number of alcohol-free months was 0.89, almost twice as many as the control group (0.42). Tables 1 and 2 illustrate the significant impact of age and gender. We used the current age and gender as control variables in the Poisson regression to analyze the rate ratio of abstinence for the whole 6-month followup and total number of alcohol-free months at the whole follow-up period in Table 3. 

 The rate ratios of stop drinking for the whole 6-month followup and total number of alcohol-free months at the whole follow-up period in Table 3 shows that the confidence interval (95% CI) of “abstinence for the whole 6-month followup” and “total number of alcohol-free months at the whole follow-up period” in the intervention group are narrow when compared to Tables 1 and 2. The narrow confidence interval of two outcomes (gender and age current variables) indicates that other variables (heavy drinking, education, born places, occupation, and marital status variables) in Tables 1 and 2 do not appear to be confounding on the main outcome variables (abstinence for the whole 6-month followup and total number of alcohol-free months at the whole follow-up period). However, the rate ratio estimation in Table 3 is similar to Tables 1 and 2. In addition, we used univariate analysis in the two groups. The result shows that the coefficient value is similar among baseline variables (Tables 1 and 2), gender, age, group variables (intervention and control groups) as Table 3, and only the group variable (intervention and control groups). That is, those variables appear to have no effect on the TGCBI intervention.

## 4. Discussion 

 This study is the first to implement TGCBI as a community intervention trial. TGCBI is a new intervention which appears to have relevance for an individual in a cultural community context. While it is acknowledged that four sessions over two months are more intensive than many brief interventions and that brief interventions are not usually delivered with such community involvement, it is contended that the interventions are still low intensity. It is evident that this relatively low intensity intervention is feasible in the Thai community—over 68% of participants adhered, as indicated by the participation of over 68% who attended all four sessions. A previous report describes the efficacy in more detail [10, 11]. 

 The two outcomes (abstinence for the whole 6-month followup and total number of alcohol-free months at the whole follow-up period) in this study support the sustainability of TGCBI intervention. Of course, limitations include the reliance on individual responses without objective confirmation (e.g., biological analyses); using the same interviewers who were not blind to the hypotheses to assess the intervention and the control groups and relatively small sample size, potentially limiting generalizability. The result of this study reported outcomes only for those participants who completed all followups in the intervention group. The results indicated that the number of participants who were for the whole 6 months and the number of alcohol-free months during followups were significantly greater (*P* < 0.05) for the intervention (*n* = 47) compared to the control group (*n* = 50). Therefore, further study will need to assess the impact in subgroups (e.g., older versus younger participants). Our study compared the TGCBI in the intervention group with no intervention in the control group. However, in the further research the two points of comparison of the effects and cost-effectiveness to those of our intervention are needed. Also, the feasibility of our intervention in other locations remains to be assessed.

In the context of these limitations, there is evidence supporting the impact of the intervention: there is good intervention adherence or compliance; the participants in the intervention are more likely to abstinent compared to the control group, and the intervention participants had more alcohol-free months compared to the controls. These two outcomes reflect the sustainability of TGCBI, at least over the 6-month followup, in the intervention community. 

The results from this study correspond with systematic reviews which have confirmed the efficacy of brief interventions in reducing risky levels of alcohol consumption in nondependent individuals [6–8, 17, 18]. A recent systematic review [19] on the efficacy of brief alcohol interventions concluded that intervention could reduce alcohol consumption. Another study on brief intervention found that it was an important and effective way to reduce alcohol-related harm, especially in primary care settings [20, 21]. This treatment strategy has been shown to be as effective for heavy drinkers as more intensive interventions, more cost-effective due to the length of treatment and can be used in a wide variety of primary care settings to reach a large number of patients. Significant reductions of up to 30% in alcohol consumption have been achieved in a variety of health care settings, including hospital and general practice [19–21]. Brief interventions in primary care are also cost-effective [22].

Our study suggests that TGCBI implementation has potential benefits as a community based intervention, and that it is a suitable tool for public health personnel in hospitals and health centers to identify alcohol use disorder, to undertake consultations, to provide accurate knowledge and understanding about alcohol consumption, and to reduce alcohol dependence in the community. In addition, we have found that brief interventions, linked to community support from elders, monks, and so forth, can be effectively delivered. In future research, we will demonstrate the impact of a cost efficient intervention that can gain support from community leaders and that seems to be readily accepted by communities. Moreover, a larger study would be necessary to undertake subgroup analyses, which clearly is merited by the importance of the findings of this initial study. Of course for those with more severe problems, who were excluded from this investigation, more intensive interventions may be required.

## 5. Conclusions

 We have presented evidence that the TGCBI has impact in terms of total abstinence and periodic abstinence. The sustainability of TGCBI beyond 6 months remains to be determined.

## Figures and Tables

**Table 1:** Effect of intervention on the rate ratios of abstinence at whole 6-month followup.

	Whether for the whole 6-month		Rate ratios	*P* value	95% CI
	followup being abstinence			
	No			
Control group	47	3	1		
Intervention group	36	11	3.62	0.050	0.99–13.13
Nonheavy drinking	46	10	1		
Heavy drinking: 5+ standards drink	37	4	0.75	0.591	0.26–2.14
Current age: 19 years old	41	2	1		
Current age: 40 years old	42	12	3.92	0.031	1.12–13.64
Male	74	10	1		
Female	9	4	2.09	0.186	0.69–6.29
Other education	33	5	1		
Primary school	50	9	1.12	0.794	0.46–2.73
Born in other provinces	14	1	1		
Born in Lopburi province	69	13	1.54	0.659	0.22–10.48
Other occupation	56	9	1		
Employee	27	5	0.73	0.587	0.24–2.23
Other marital status	47	3	1		
Married status	36	11	0.88	0.809	0.31–2.43

**Table 2:** Effect of intervention on number of alcohol-free months at the whole 1, 3, and 6 months at the whole follow-up period.

	Number of alcohol-free months at the whole 1, 3, and 6 months	Rate ratios	*P* value	95% CI
Control group	21	1		
Intervention group	42	2.1	0.040	1.03–4.3
Non heavy drinking	46	1		
Heavy drinking: 5+ standards drink	17	0.63	0.250	0.29–1.36
Current age: 19 years old	14	1		
Current age: 40 years old	49	2.41	0.033	1.07–5.42
Male	46	1		
Female	17	2.03	0.047	1.00–4.09
Other education	22	1		
Primary school	41	1.11	0.733	0.58–2.12
Born in other provinces	8	1		
Born in Lop Buri province	55	1.02	0.959	0.4–2.61
Other occupation	42	1		
Employee	21	0.73	0.445	0.34–1.6
Other marital status	20	1		
Married status	43	0.85	0.643	0.43–1.66

**Table 3:** Effect of intervention, the rate ratios of abstinence for the whole 6-month followup and total number of alcohol-free months at the whole follow-up period among risky drinkers in two high prevalence communities.

	Abstinence for the whole		Total number of alcohol-free months	
	6-month followup		at the whole follow-up period	
	Rate ratios	95% CI	Rate ratios	95% CI
Control group	1		1	
Intervention group	3.49	1.07–11.37	1.95	1.00–3.79
Current age: 19 years old	1		1	
Current age: 40 years old	4.08	0.98–17.01	2.47	1.12–5.43
Male	1		1	
Female	2.06	0.74–5.75	2.04	1.06–3.92
